# Investigating cellular heterogeneity at the single-cell level by the flexible and mobile extrachromosomal circular DNA

**DOI:** 10.1016/j.csbj.2023.01.025

**Published:** 2023-01-24

**Authors:** Jiajinlong Kang, Yulin Dai, Jinze Li, Huihui Fan, Zhongming Zhao

**Affiliations:** a Center for Precision Health, School of Biomedical Informatics, The University of Texas Health Science Center at Houston, Houston, TX 77030, USA; b MD Anderson Cancer Center UTHealth Graduate School of Biomedical Sciences, Houston, TX 77030, USA; c Department of Epidemiology, Human Genetics, and Environment Sciences, School of Public Health, The University of Texas Health Science Center at Houston, Houston, TX 77030, USA; d Department of Neurology, McGovern Medical School, The University of Texas Health Science Center at Houston, Houston, TX, USA; e Human Genetics Center, School of Public Health, The University of Texas Health Science Center at Houston, Houston, TX 77030, USA

**Keywords:** Single-cell ATAC sequencing, Glioblastoma, Ec/eccDNA, Distal regulator, Transregulation

## Abstract

Extrachromosomal circular DNA (eccDNA) is a special class of DNA derived from linear chromosomes. It coexists independently with linear chromosomes in the nucleus. eccDNA has been identified in multiple organisms, including Homo sapiens, and has been shown to play important roles relevant to tumor progression and drug resistance. To date, computational tools developed for eccDNA detection are only applicable to bulk tissue. Investigating eccDNA at the single-cell level using a computational approach will elucidate the heterogeneous and cell-type-specific landscape of eccDNA within cellular context. Here, we performed the first eccDNA analysis at the single-cell level using data generated by single-cell Assay for Transposase-Accessible Chromatin with sequencing (scATAC-seq) in adult and pediatric glioblastoma (GBM) samples. Glioblastoma multiforme (GBM) is an aggressive tumor of the central nervous system with a poor prognosis. Our analysis provides an overview of cellular origins, genomic distribution, as well as the differential regulations between linear and circular genome under disease- and cell-type-specific conditions across the open chromatin regions in GBM. We focused on some eccDNA elements that are potential mobile enhancers acting in a trans-regulation manner. In summary, this pilot study revealed novel eccDNA features in the cellular context of brain tumor, supporting the strong need for eccDNA investigation at the single-cell level.

## 1 Introduction

Eukaryotic DNA typically exists in multiple pairs of linear chromosomes. However, there is a special class of DNA, extrachromosomal circular DNA (eccDNA), that is derived from linear chromosomes and exists independently in the nucleus [1]. Since its first discovery in plants in 1965 [2], eccDNAs have been identified in more organisms including *Drosophila* [3], mouse [4], and humans [5]. Based on their length and functions, eccDNAs are currently grouped into four categories: 1) small polydispersed circular DNA (spcDNA) (100 bp-10 kb), 2) telomeric circles (integral multiples of 738 bp), 3) microDNA (100–400 bp), and 4) extrachromosomal DNA (ecDNA) (1–3 Mb) [6]. In human tumors, ecDNAs tend to contain entire oncogenes and are often involved in gene amplification, which is related to carcinogenesis and drug resistance. For example, it has been shown that ecDNA amplifies *N-MYC* in neuroblastoma, *EGFR* in glioblastoma, and *HER2* in breast cancer [4]. In addition to oncogene amplification, shorter eccDNAs (<100 kb) may harbor regulatory elements such as distal enhancers, which serve as mobile regulators and provide more extensive trans-regulations in an unforeseen and flexible way. It has been reported that microDNA may express functional small regulatory RNAs that interfere with gene expression [7]. Furthermore, recent studies confirm that longer eccDNAs (1–3 Mb) could also have regulatory functions by enhancer hijacking through co-amplification of proximal enhancers of an oncogene [8] or by acting as mobile enhancers [9].

Considering the diversity and functional importance of eccDNA in tumorigenesis, understanding of cellular regulation involving eccDNA is currently lacking due to the absence of comprehensive characterization at both bulk tissue and single-cell level. Apart from traditional imaging methods such as optical microscopy and electron microscopy, various computational tools have been developed to capture eccDNA using sequencing data. These tools include AmpliconArchitect [10], Circle_Map [11], Circle_finder [12], CIDER-seq2 [13], ECCsplorer [14] and ecc_finder [15]. These tools were built and evaluated using bulk sequencing data, such as whole-genome sequencing (WGS). Most recently, Assay for Transposase‐Accessible Chromatin with high‑throughput sequencing (ATAC‑seq) data have also been adapted based on the fact that eccDNAs frequently harbor genomic regions open for regulations [16]. So far, for the sequencing data generated without the procedure to enrich circular DNA molecules, investigators often computationally detect eccDNAs based on their characteristics [16]. Specifically, detection of eccDNAs using ATAC-seq relies on abnormally mapped reads consisting of split reads (reads mapped to two distinct locations on the reference genome) and discordant reads (paired-reads facing outward on the reference genome) that are generated when Tn5 inserts adaptors onto eccDNA either close to or far away from the junction sequence.

Compared to the WGS approach, ATAC-seq presents a more convenient and effective way of building the eccDNA landscape at a larger scale. Despite the rapid advancement of eccDNA research, previous studies have mainly focused on using bulk sequencing, which cannot detect the cell-type-specific regulatory landscape of eccDNA during tumorigenesis. Here, we extended the scope of eccDNA research into the single-cell level, hoping to gain the true biological resolution to decode the additional cellular regulatory heterogeneity introduced by eccDNA, and to encourage eccDNA-centered studies in human cancers.

## 2 Results

### 2.1 Genomic view of eccDNA in GBM samples at the single-cell level

eccDNA has been observed in both ATAC-seq and WGS in glioblastoma multiforme (GBM) [16]. GBM is composed of diverse cell types with a vast amount of intra- and inter-tumor heterogeneity. While GBM is more prevalent in adults, pediatric GBM does occur and accounts for about 15% of all pediatric brain tumors [17]. There are well-documented differences between adult and pediatric GBM, for example the variation in innate and adaptive immune invasion leads to a less immunosuppressive microenvironment in pediatric GBM [17]. The publicly available scATAC-seq data for GBM thus provides a valuable resource for us to mine potential eccDNAs at the single-cell level.

**Fig. 1:**
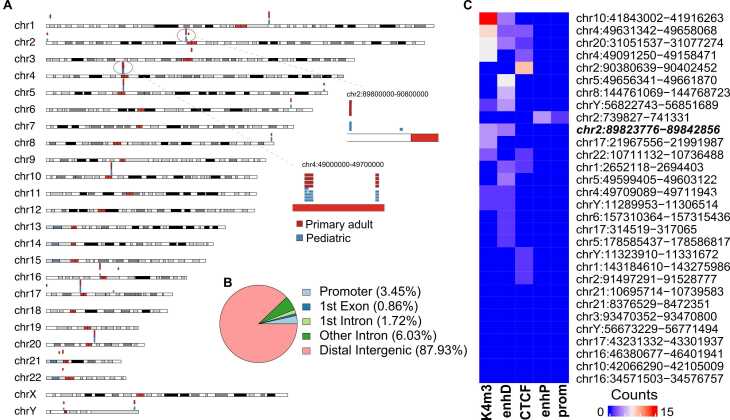
**The overview of eccDNAs at the single-cell level in pediatric and adult glioblastomas (GBMs).** (**A**) Karyoplot displaying eccDNA locations on linear chromosomes. Two zoom-in sections demonstrate eccDNAs detected in GBMs (sample group: red, primary adult GBMs; blue, pediatric GBMs). Cytoband colors in the karyoplot: Red, centromeric; white, Giemsa negative bands; different shades of grey and black, Giemsa positive bands. Darker shades of grey indicate higher intensity of Giemsa positivity. (**B**) Pie chart summarizing genomic distribution of eccDNAs from all samples combined. (**C**) Heatmap showing overlapping regulatory elements such as enhancers with eccDNAs. Frequencies are colored in gradient scales, ranging from blue (low frequency) to red (high frequency). A repetitive eccDNA origin shared across different samples and embedding distal enhancer structures (chr2:89823776−89842856) is highlighted in bold and italic font. Abbreviations: K4m3, DNase-H3K4me3; enhP, proximal enhancer-like signature; CTCF, CCCTC-binding factor; enhD: distal enhancer-like signature; prom, promoter-like signature.

We collected and integrated scATAC-seq dataset for a total of nine GBM samples [18], [19], including five pediatric and four adult GBM samples. Starting from the raw sequencing reads, we applied the state-of-the-art ecc_finder algorithm to identify inappropriately mapped reads, i.e., split and discordant reads (see Materials and Methods; also SF 1). The sample-based eccDNA catalogue is shown in Table 1, with their aligned linear chromosome locations illustrated in Fig. 1A. Overall, the identified eccDNAs were scattered across the genome, and were commonly shared between adult and pediatric GBM samples. We also observed similarities in eccDNA length across the samples within each of the two groups (adults and pediatric). More heterogeneous patterns in both length and location distribution were found in pediatric GBM (Fig. 1A, zoom-in sections on chromosomes 2 and 4). Thirty common eccDNAs were identified when we chose the union of overlapping eccDNA regions shared across all the samples. We found that these eccDNAs were mostly short in length (ranges: 306–145,754 bp) and mainly enriched in distal intergenic regions (Fig. 1B; 87.93%).

**Table 1:** Sample-based eccDNA catalogue.

SRA	Status	eccDNA	Percentage of cells harboring eccDNA
SRR10315835	Adult primary glioblastoma tissue	chr1:143184615–143275868; chr10:41843349–41916253; chr16:34571510–34576756; chr17:21968723–21991976; chr2:89825156–89841143; chr20:31051578–31076467; chr4:49091284–49157869; chr4:49631387–49658060; chr5:49599456–49603119; chr5:49656346–49661867; chr6:157310412–157315333; chrY:11323910–11331672; chrY:56673236–56771486	9.96%
SRR10315836	Adult primary glioblastoma tissue	chr1:143184614–143275894; chr1:2652118–2684542; chr10:41843212–41916258; chr16:34571503–34576757; chr17:21967556–21991976; chr17:314519–317065; chr2:89823883–89841143; chr20:31051537–31077112; chr21:10700507–10739583; chr3:93470352–93470800; chr4:49091262–49158469; chr4:49631349–49658068; chr4:49709089–49711938; chr5:49599427–49603116; chr5:49656342–49661867; chrY:56822743–56851689	15.19%
SRR10315837	Adult primary glioblastoma tissue	chr1:143184612–143275983; chr1:2682915–2694403; chr10:41843231–41916263; chr16:34571506–34576757; chr16:46380677–46401941; chr17:21968716–21991986; chr2:739827–741112; chr2:89825266–89842856; chr2:91497291–91528777; chr20:31051537–31077274; chr21:10695714–10738318; chr21:8376529–8472351; chr22:10711132–10736488; chr4:49091252–49157869; chr4:49631351–49658067; chr4:49709089–49711943; chr5:178585437–178585743; chr5:49599405–49603122; chr5:49656346–49661870; chr6:157310364–157315164; chr8:144767333–144768654; chrY:11290910–11306500	13.96%
SRR10315838	Adult primary glioblastoma tissue	chr1:143184610–143275984; chr10:41843002–41916248; chr16:34571510–34576756; chr17:21968723–21991977; chr2:89823776–89841183; chr20:31051539–31077009; chr3:93470352–93470800; chr4:49091250–49157181; chr4:49631364–49658068; chr5:49656350–49661870; chrY:56673233–56771492	15.71%
SRR13282530	Patient-derived pediatric glioblastoma tissue (relapse)	chr1:143184614–143275951; chr10:41843226–41916248; chr17:21968743–21991975; chr2:90380639–90402452; chr20:31051540–31076465; chr4:49091289–49156577; chr4:49631401–49658065; chr5:49656348–49661868	9.20%
SRR13282531	Patient-derived pediatric glioblastoma tissue (relapse)	chr10:41857290–41916225; chr17:21968781–21991569; chr2:739916–741331; chr2:89826160–89841128; chr20:31051563–31076456; chr4:49091302–49156541; chr4:49631408–49658048; chr5:49656348–49661857; chr6:157310421–157315382; chr8:144766838–144768598; chrY:56828779–56840003	10.11%
SRR13282532	Patient-derived pediatric glioblastoma tissue (relapse)	chr1:143260441–143268678; chr10:41857287–41871986; chr10:41873065–41915909; chr17:21968778–21991897; chr17:43231332–43301937; chr2:89826161–89841128; chr20:31051539–31076455; chr4:49091387–49121313; chr4:49122351–49156537; chr4:49631407–49658048; chr4:49709167–49711912; chr5:49599433–49603099; chr6:157310423–157315436; chr8:144767275–144768500	8.79%
SRR13320479	Primary patient-derived pediatric glioblastoma	chr1:143184612–143275986; chr10:41843027–41916258; chr10:42066290–42105009; chr16:34571510–34576756; chr17:21968718–21991987; chr2:89824986–89841138; chr20:31051537–31077112; chr4:49091264–49158471; chr4:49631342–49658068; chr5:178585544–178586817; chr5:49599412–49603119; chr5:49656341–49661870; chrY:11289953–11306514; chrY:56673229–56771494; chrY:56825444–56851466	14.51%
SRR13320481	Primary patient-derived pediatric glioblastoma	chr10:41873325–41881847; chr20:31051592–31060773; chr20:31061789–31069767; chr4:49091369–49112948; chr5:49656411–49661832; chr8:144761069–144768723	2.56%

### 2.2 Majority of the identified eccDNAs harbor enhancers

Our identified eccDNAs are largely overlapped with the intergenic regions. The relationship between these regions and distal regulators such as enhancers was thus investigated. We downloaded the ENCODE candidate cis-regulatory elements combined from all cell types [20], and compared them with our eccDNAs. We discovered that 22 of the 30 eccDNAs overlapped with at least one cis-regulatory element; these elements included promoters (column names as prom and K4m3), distal enhancers (enhD), proximal enhancers (enhP), and insulator CCCTC-binding factor (CTCF) (Fig. 1C). These findings suggested that although these eccDNAs are not long enough to harbor whole oncogenes, they might operate by acting as mobile functional regulators independently from their counterparts on the linear chromosomes.

### 2.3 Single-cell analysis reveals disease-specific and cell-type-specific regulatory roles of eccDNA

**Fig. 2:**
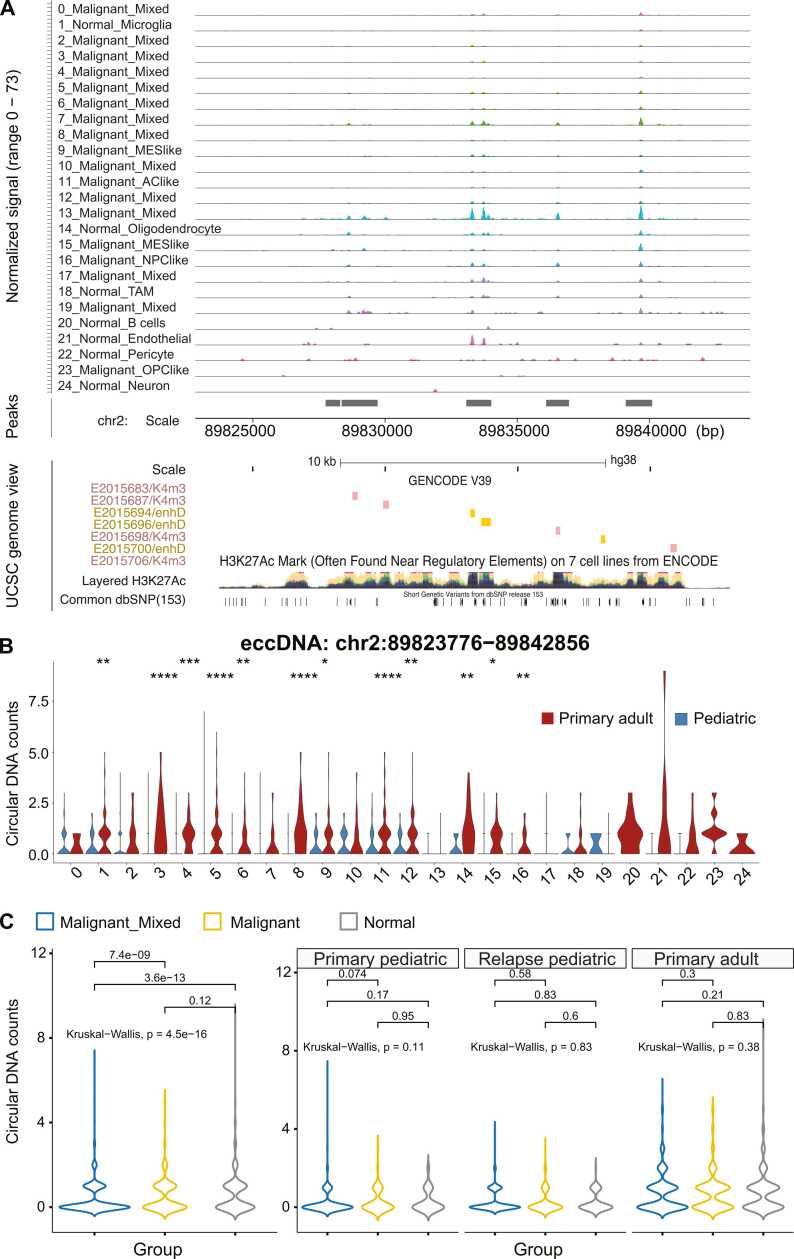
**Single-cell analysis revealed cell type-specific coverage of eccDNA chr2:89823776−89842856.** (**A**) Linear read coverage view (top track; each row represents a Seurat cell cluster) for eccDNA chr2:89823776−89842856, together with track view of peaks (middle) and UCSC Genome Browser view (bottom). Overlapping regulatory elements are colored by annotation sources, together with layered H3K27ac histone signal for enhancers shown in the bottom track. Abbreviations: TAM: tumor-associated macrophage; NPClike, neural-progenitor-like; OPClike, oligodendrocyte-progenitor-like; AClike, astrocyte-like; MESlike, mesenchymal-like. (**B**) Circular coverage of eccDNA at locus chr2:89823776−89842856 in GBM samples. Seurat cell clusters are named in the format of [Cluster ID]_[Tumor or normal cell]_[cell type or cell states]. Mixed: the malignant cluster is a mixture of at least two cell states out of MESlike, OPClike, NPClike and AClike. The non-parametric Wilcox test was applied to compare eccDNA distributions by cell type between adult and pediatric conditions. Significance: ****: p < 0.0001. ***: p < 0.001. **: p < 0.01. *: p < 0.05. (**C**) Comparison of eccDNA chr2:89823776−89842856 between malignant and normal cells. Left penal: comparison of the eccDNA distribution across groups of malignant, malignant_mixed and normal cells. Right penal: the left penal comparison was further stratified by tumor sample condition. The non-parametric Kruskal-Wallis test was used to test whether the three cell groups were originated from the same distribution.

To further elucidate the regulatory role of these eccDNAs, we increased the resolution of analysis to the single-cell level and successfully co-mapped the eccDNA reads to condition- and cell type-specific signatures. By integrating our in-house approach of cell-barcode tracing strategy as well as routine quality control of cell filtering [21], we successfully parsed the split and discordant reads at the single-cell level. Among the 10,569 cells we analyzed, we found 3475 cells containing at least one eccDNA that satisfied all of the routine CellRanger [22], Signac [21], and Seurat [23] processing pipelines (SF 1). To accurately label cell types in our integrated samples, we cross-referenced our scATAC-seq dataset with four independent well-annotated scRNA-seq GBM datasets (see Materials and Methods). Malignant cells were further stratified into a four-state paradigm [24], which included neural-progenitor-like (NPC-like), oligodendrocyte-progenitor-like (OPC-like), astrocyte-like (AC-like), and mesenchymal-like (MES-like) tumor cells. However, the majority of the malignant cell clusters were identified as hybrids mapped to more than two cellular states mentioned above. This further demonstrated the vast intra-tumor heterogeneity in GBM (Fig. 2A, row labels; SF 2).

An example of eccDNA (chr2:89823776−89842856, Fig. 1C, highlighted label) is illustrated along with cell-type-specific linear coverage (Fig. 2A, top track, coverage determined using properly mapped reads), as well as overlapping features such as identified ATAC peaks (Fig. 2A, middle) and distal regulatory enhancers from ENCODE (Fig. 2A, bottom). As shown, malignant cell cluster #13 and endothelial cell cluster #21 had stronger linear signals of this eccDNA. However, when we examined the circular coverage of the same eccDNA (coverage determined using discordantly mapped reads) across different cell clusters more closely, the circular signal of this region did not appear in malignant cell cluster #13 but appeared in endothelial cell cluster #21 (Fig. 2B). This indicated a cell-type-specific regulatory mechanism of eccDNA.

Interestingly, eccDNA chr2:89823776−89842856 is also located in the vicinity (within the distance from 8935 to 391,956 bp) of a group of VDJ-recombination genes on the linear chromosome 2. As demonstrated in Fig. 2A, B cell cluster #20 did not yield sufficient linear coverage in this region; however, we observed an exclusive and dominant circular coverage of eccDNA derived from this linear location (Fig. 2B). According to a previous report that eccDNA is a potent stimulant of immune response in dendritic cells/macrophages [25] and participates in the tumor proinflammatory response [26], we hypothesized that VDJ recombination in B cells within the primary GBM microenvironment could be potentially exclusively regulated by this eccDNA. Note that the enhancer activity shown on the linear chromosome does not necessarily translate into enhancer activity on the circular form of this eccDNA. Therefore, further experiments are needed to confirm whether there is a direct interaction between this eccDNA and the nearby VDJ-recombination genes on the linear chromosome. Furthermore, the differential regulation potentially initiated by this eccDNA is consistently observed in several other malignant cell clusters, for instance, 7 cell clusters (#0 to #6), as shown in the linear coverage (Fig. 2A), and the disease- and cell-type-specific circular coverage (Fig. 2B; SF 3). A closer examination on the eccDNA distributions across samples of different malignancy status indicated significant differences when comparing the malignant and normal cell groups with the malignant mixed group (Fig. 2C, left panel). No significance was found between the malignant and normal cell groups, or between any two cell groups in each sample group (Fig. 2C, right panel).

Altogether, this mosaic nature of eccDNA distribution across various cell types in human tumors cannot be elucidated using bulk sequencing data. More in-depth genome-wide studies characterizing eccDNA in human cancers are in pressing need to yield a better understanding of their contributions to tumorigenesis.

### 2.4 Conclusion and discussion

Our computational approach revealed the landscape of eccDNAs in human GBM. It not only demonstrated the feasibility of detecting and studying eccDNAs at the single-cell level, but also provided better biological resolution when compared to previous eccDNA studies using bulk RNA sequencing. By integrating scATAC-seq data from nine adult and pediatric GBM samples, we provided an atlas view of the cellular origin, the aligned linear genomic distribution as well as differential regulation between linear and circular form under disease- and cell-type-specific conditions across the open chromatin regions in GBM.

Downstream analysis led us to further investigate the extensive overlap between eccDNA-mapped linear genomic locations and distal regulatory elements. Our results highlighted that the majority of our identified eccDNAs (i.e., 22 out of 30, 73.33%) originated from the linear chromosomes where at least one distal regulator was present, for example enhancers and/or insulators. Previous studies demonstrated that long eccDNAs (ranging from a few hundred kb to several Mb) could harbor whole genes and, therefore, they act as an amplifier of oncogenes involving tumorigenesis and drug resistance [27]. In contrast, short eccDNAs with length of tens of kb tended to be immunostimulant in tumor-infiltrating immune cells due to their circular structure instead of specific sequences [25]. Our results expanded the understanding of eccDNA functions in both tumor cells and the cells within tumor microenvironment, and with a specific focus on short eccDNAs. Our study provided evidence in that short eccDNAs could harbor biologically meaningful sequences such as regulatory elements acting as mobile enhancers. This function is supported by the most recent discoveries on the potential functions of eccDNAs during tumorigenesis, namely that eccDNAs with length of 1–3 Mb might function as mobile enhancers to globally interfere with chromosomal transcription [9]. Zhu et al. also provided convincing evidence that direct interactions between eccDNAs and linear chromosomes could occur through the RNA polymerase II-mediated eccDNA-chromatin complex. However, whether the same mechanism applies to shorter eccDNAs remains an open question.

Collectively, our results suggested that eccDNA might act as mobile regulators, thus contributing to the vast amount of heterogeneity observed in, but not limited to, human GBMs. Other forms of circular nucleic acid sequences (e.g., circular RNAs) have been extensively studied for functional role in a variety of tissues and tumorigenesis. This study of eccDNA sheds light on their potential roles in cellular function and cancer biology [28], [29]. Specifically, further experiments are warranted to elucidate the detailed roles of eccDNA during tumorigenesis.

By comparing the eccDNA distributions across multiple groups, we observed a higher number of locus-specific eccDNAs inferred in the adult than pediatric GBM samples. eccDNAs were previously reported as apoptotic products [25]; accordingly, this condition-specific difference in eccDNA could potentially reflect a stronger genome instability in adult than pediatric GBMs. By showcasing one eccDNA mapped to chromosome 2, we hypothesized that eccDNAs might regulate tumor’s immune microenvironment by impacting VDJ recombination in local B cells. However, the causal relationship between this specific eccDNA and VDJ recombination is uncertain. VDJ recombination is a process involving frequent structural rearrangements, which could potentially increase the probability of generating sequence deletion, circularization, and shaping of eccDNAs. Therefore, we cannot rule out the possibility that this eccDNA, which potentially functions as a mobile regulator, is a byproduct of VDJ recombination. Further validations are needed to accurately dissect their relationship.

Remarkably, the eccDNAs identified in this study were largely mapped to intergenic regions, which is somewhat contradictory to the previous reports [30], [31] of over 50% of detected eccDNAs in the genic or pseudogenic regions. Several factors may contribute to this difference in mapping the short reads to functional genomic regions: sequencing technologies (WGS or partial-genome such as ATAC-seq) and sequence depth, eccDNA-inferring algorithms, and tissue and disease conditions. Of note, the previous studies [30], [31] used normal tissues, whereas GBM tumor samples were investigated in this study.

The vast majority of cells (67.77%) harboring eccDNAs were filtered out by the routine processing pipeline when considering all relevant quality control metrics. As eccDNAs are potentially generated during apoptotic process [25], it is possible that those eccDNA-harboring cells filtered out could exhibit stronger level of apoptosis comparing to the ones that passed quality control. Intriguingly, this group of filtered-out cells likely vulnerable to procedural stress during single-cell sequencing could potentially harbor highly informative eccDNA profile. It also raises the question whether it is suitable to profile eccDNAs at the single-cell resolution and calls for a nearly stress-free single-cell sequencing platform to maximally capture viable cells that mimic the living cells in-vivo.

In addition, the low detection rate of eccDNAs in our study, as indicated that 67.77% of the cells harboring eccDNAs were filtered out during quality control, could also result from the high drop-out rates and data sparsity due to the loss of DNA material in the scATAC-seq protocol [32]. Thus, it is necessary to supplement this approach with genomic sequencing to increase detection coverage. The comprehensive catalogue of eccDNA during tumorigenesis is emerging. Cells harboring different types of eccDNAs may contribute to the diverse phenotypes observed in GBM. Diverse phenotypes confer specific survival advantages and, thus, benefit the evolutionary process of different cell subpopulations in both tumor and tumor-associated environments.

There are several main limitations in our study. The length of eccDNAs we identified was short in general, ranging from tens of kb to ∼150 kb, which was a potential bias of the eccDNA detection tool chosen in this study. When we learn more eccDNA structures and how they affect the mapping process of sequencing reads, better rules to define the circular structure will evolve accordingly. In particular, ecc_finder initially identifies eccDNAs longer than 1 Mb. However, the subsequent stringent quality control step that requires an even distribution of split and discordant reads accidently throws out all the long hits, because the middle portions of these eccDNAs lack sufficient reads to undergo peak naming with stringent false discovery rates. We propose a potential workaround in the future which loosens the above assumption by focusing on split reads only and manually tracking breakpoints spanning several Mb in the reference genome. Quality control measures remain to be further investigated, since candidate long eccDNAs tend to be false positives. In addition to algorithm limitations, representation of long-read eccDNAs in our study is limited by the current sequencing approach. A more efficient approach is to eliminate or reduce linear genomes in the library preparation using exonuclease, so that circular sequences will be enriched [25]. To ensure the generation of high-confident consensus sequences that match the full length of eccDNAs, sample processing could be coupled with long-read sequencing (e.g., Nanopore or PacBio) of individual eccDNAs after cell amplification.

Taken together, our study presents a novel approach to study eccDNA at the single-cell level, which also yields novel insights into the potential regulatory roles of eccDNA in tumorigenesis from both disease- and cell-type-specific perspectives. This has the potential to expand our current understanding of eccDNA based on bulk sequencing approaches. Given the presence of eccDNA in both tumor and tumor microenvironment, and its role associated with tumorigenesis and drug resistance, we expect the refinement of currently available methods to extend eccDNA research. One promising avenue is the rapid development of new algorithms specifically targeting eccDNA at the single-cell level to gain more coverage in the near future.

## 3 Materials and methods

### 3.1 Single-cell ATAC sequencing (scATAC-seq) data curation, processing, and annotation

A total of nine GBM samples profiled by scATAC-seq [18], [19] (GEO accession IDs: GSE139136, GSE163655, and GSE163656), including five pediatric and four adult GBM samples, were downloaded and curated (SF 1, Step 1; ST 1). Starting from the raw sequencing reads, all samples were preprocessed, quality-controlled and integrated using the standard CellRanger mapping against the GRCh38 reference genome (Version 1.1.0, 10x Genomics) [29], [33], Seurat and Signac pipelines with default settings (SF 1, Step 2) [30]. Specifically, quality control was performed based on the following inclusion criteria: the nucleosome signal (NS) score < 4, transcription start site (TSS) enrichment score ≥ 1, the ratio of reads mapped to peaks ≥ 15, the fraction of fragments mapped to blacklisted genomic regions < 5%, and the total number of fragments in peaks between 10th and 90th percentile per sample. Cells failing quality control were filtered out from further analysis. To label cell types, the integrated scATAC-seq dataset was cross-referenced with four independent well-annotated scRNA-seq GBM datasets, including GSE84465 [22], GSE131928 [23], GSE138794 [24] and GSE151506 [25]. Cell labels were confidently transferred from scRNA-seq to scATAC-seq data using R package Signac if the same cell label was nominated by at least two out of four scRNA-seq datasets. In addition to broad classes of malignant and normal cell clusters, malignant cells were further stratified into a four-state paradigm [23], which included neural-progenitor-like (NPC-like), oligodendrocyte-progenitor-like (OPC-like), astrocyte-like (AC-like), and mesenchymal-like (MES-like) cell states using R package scrabble based on cell-state-specific signatures.

### 3.2 eccDNA identification and downstream analysis

The algorithm ecc_finder [15] was applied to identify split and discordant reads using paired-end short-reads mapping mode (SF 1, Step 3). Reads used to identify eccDNAs were then traced back using single cell barcoding system to their originated cells. A count by cell matrix for eccDNAs was therefore generated. Common eccDNAs were defined as the union of overlapping eccDNAs across all the samples. Next, their circular and linear coverage were calculated (SF 1, Step 4). An overview of the eccDNA along chromosomes was visualized in a karyoplot using R package karyoploteR (SF 1, Step 5). Genomic annotations (promoter, 1st exon, 1st intron, other introns, or distal intergenic regions) of eccDNAs was done using R package ChIPseeker. To investigate whether eccDNA overlaps with distal regulators, ENCODE cCREs annotation file (ENCODE candidate cis-regulatory elements combined from all cell types; human genome GRCh38) was downloaded using UCSC table browser [20]. Heatmap was plotted using R package ComplexHeatmap (SF 1, Step5).

### 3.3 Statistical analysis

Group-wise comparisons were carried out using non-parametric Wilcoxon Rank Sum and Signed Rank tests to calculate the statistical significance.

## Funding

This work was partially supported by 10.13039/100000002National Institutes of Health (NIH) grant (R01LM012806) and the 10.13039/100004917Cancer Prevention and Research Institute of Texas grant (CPRIT RP180734 and RP170668). ZZ was also partially supported by 10.13039/100000002NIH grants R03AG077191 and R01DE030122. JK is a CPRIT Predoctoral Fellow in the Biomedical Informatics, Genomics and Translational Cancer Research Training Program (RP210045).

## Author’s contributions

HF and ZZ conceived the study. HF, JK, YD and JL collected the data, performed the data analysis and interpreted the results. JK, HF and ZZ wrote the manuscript. All authors read and approved the final manuscript.

## Compliance with ethical standards

None.

## Declaration of interest

The authors declare no conflict of interest.
