# Supplementary figures and images for: Investigating cellular heterogeneity at the single-cell level by the flexible and mobile extrachromosomal circular DNA
Source: Comput Struct Biotechnol J. 2023 Jan 24;21:1115–21. doi: 10.1016/j.csbj.2023.01.025 (PMC9900259; doi:10.1016/j.csbj.2023.01.025)

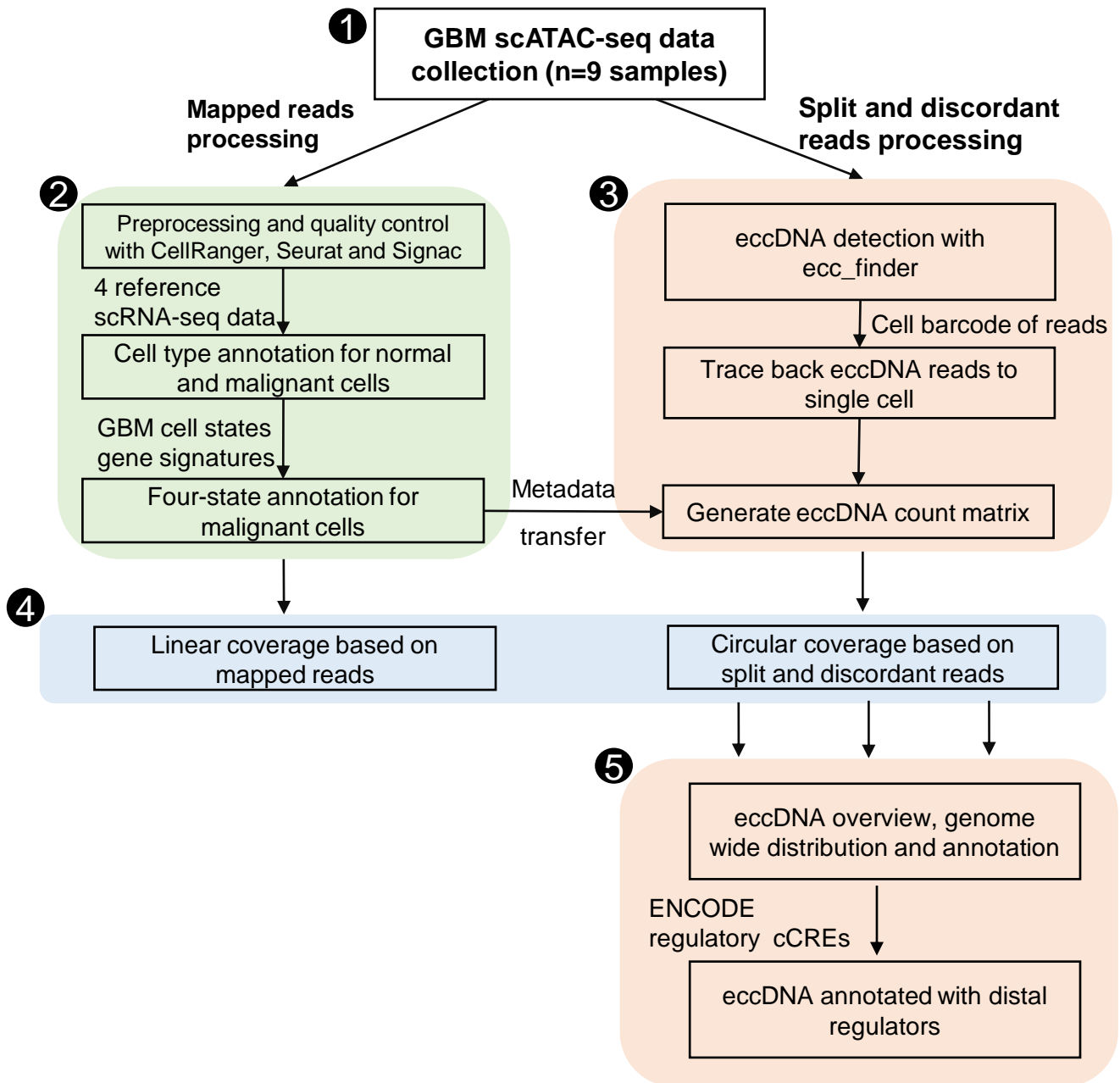

Supplement: Supplementary file 1 — Supplementary material [file mmc1.pdf]

**A**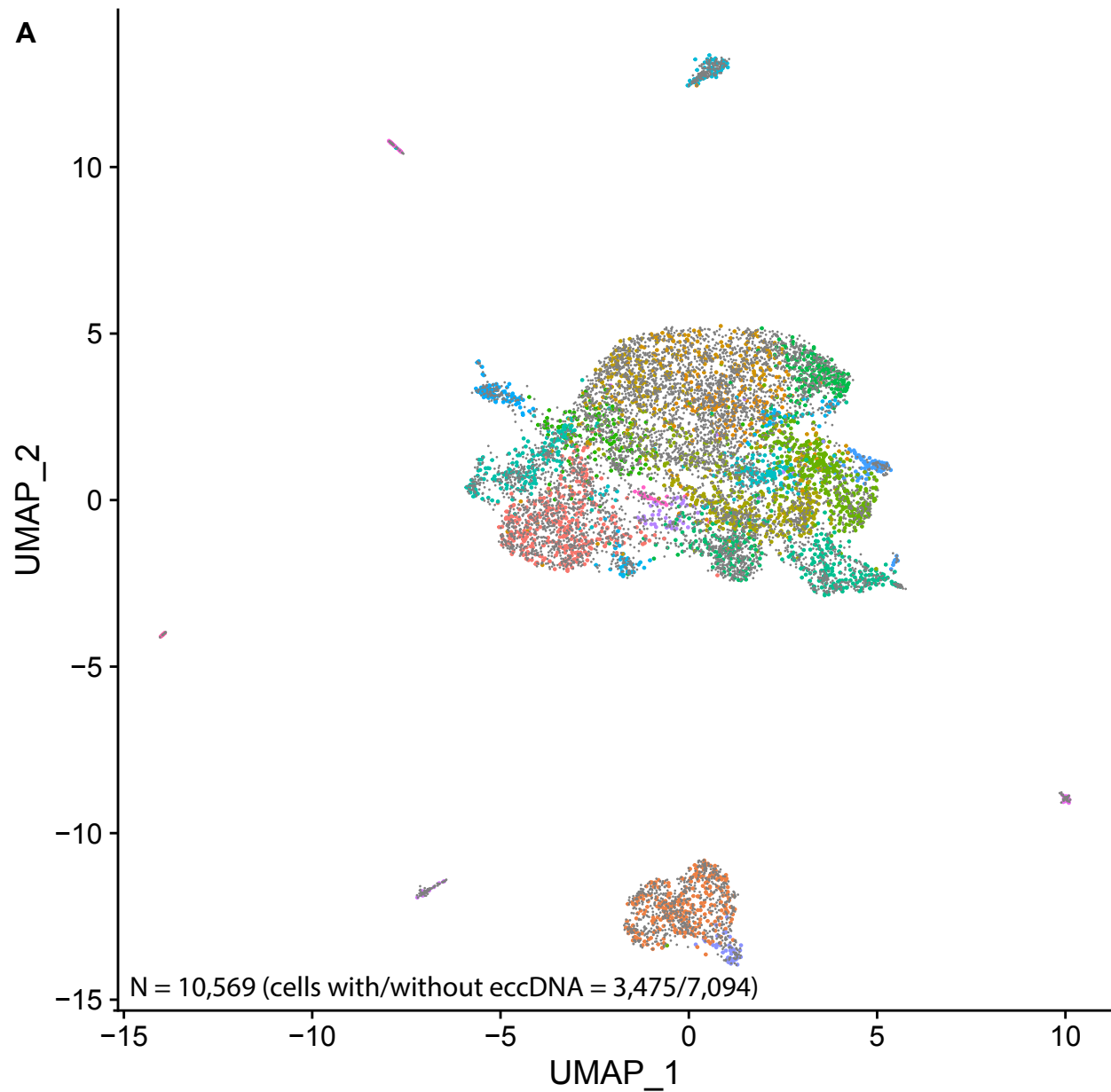**B**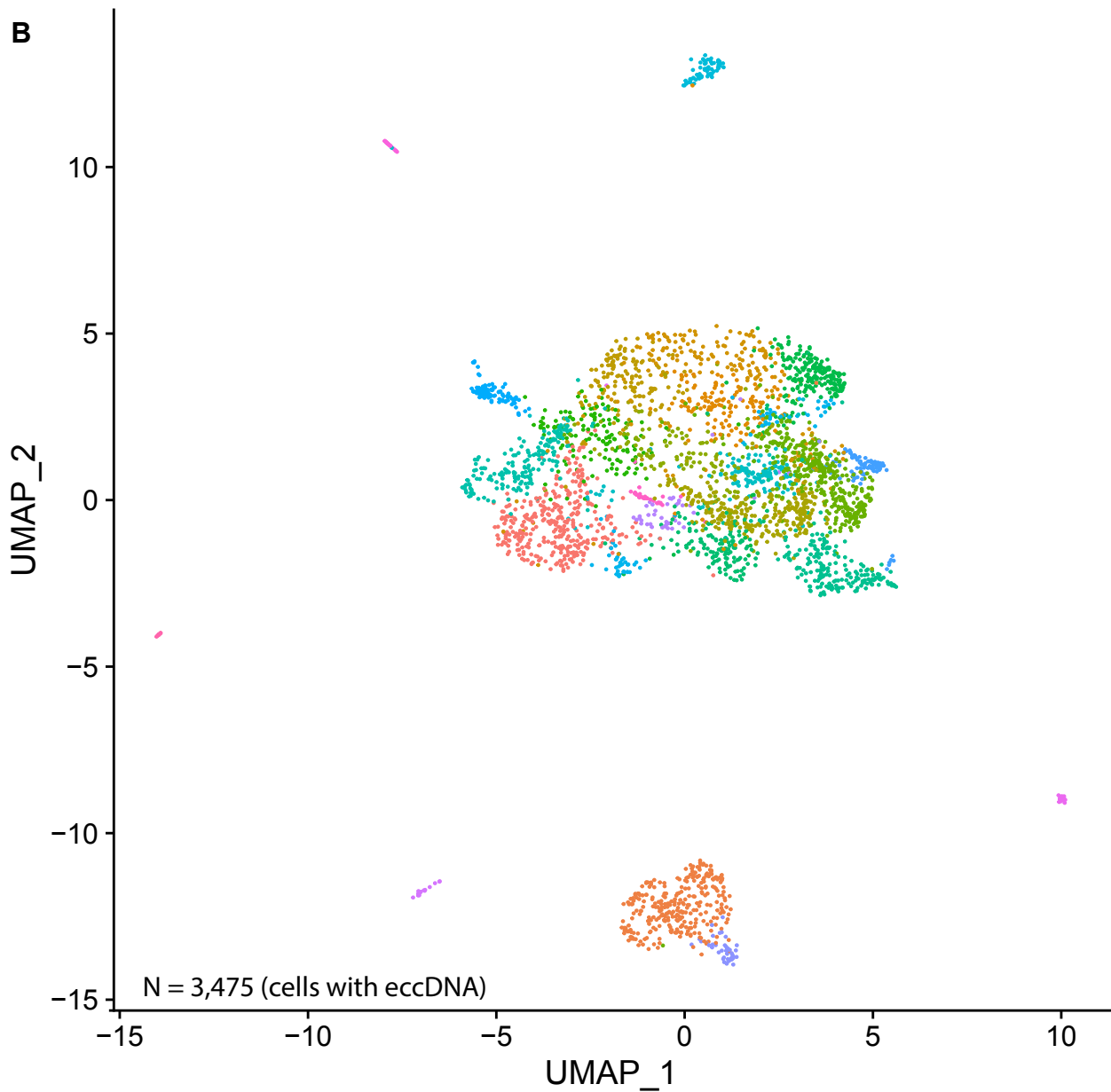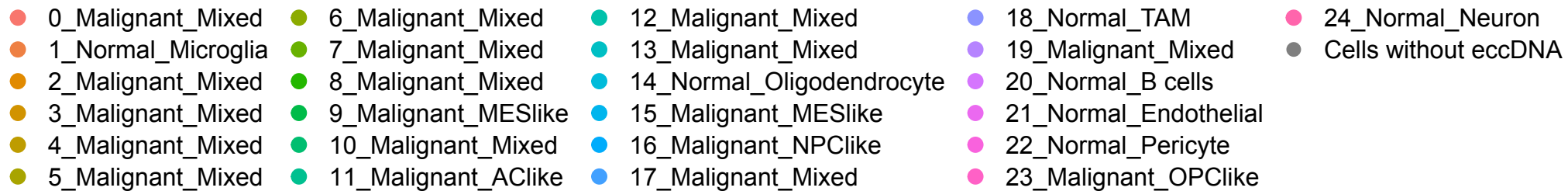

Supplement: Supplementary file 2 — Supplementary material [file mmc2.pdf]

# chr2-89823776-89842856

A

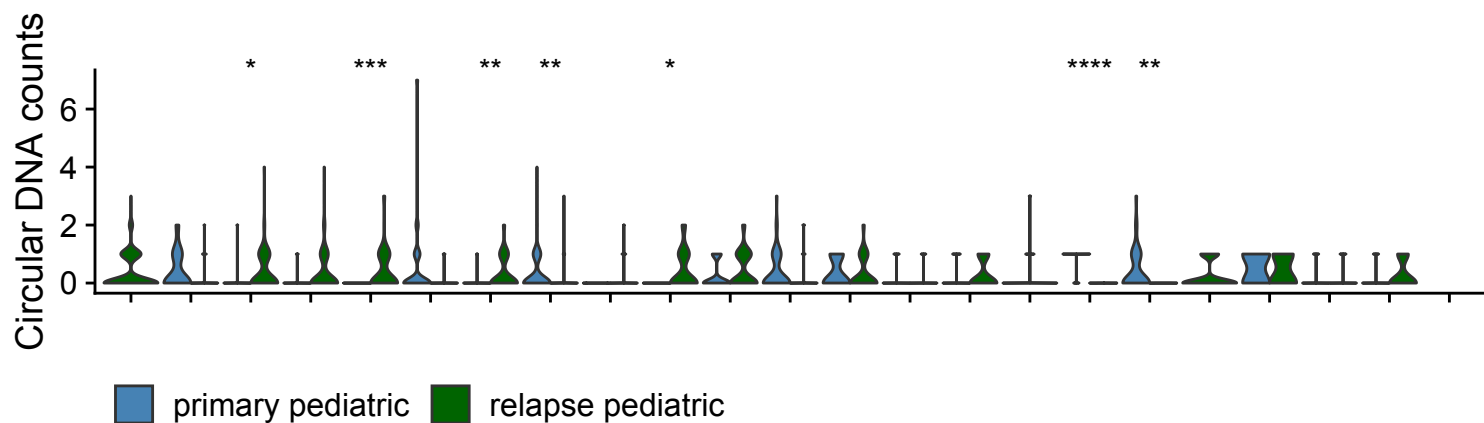

B

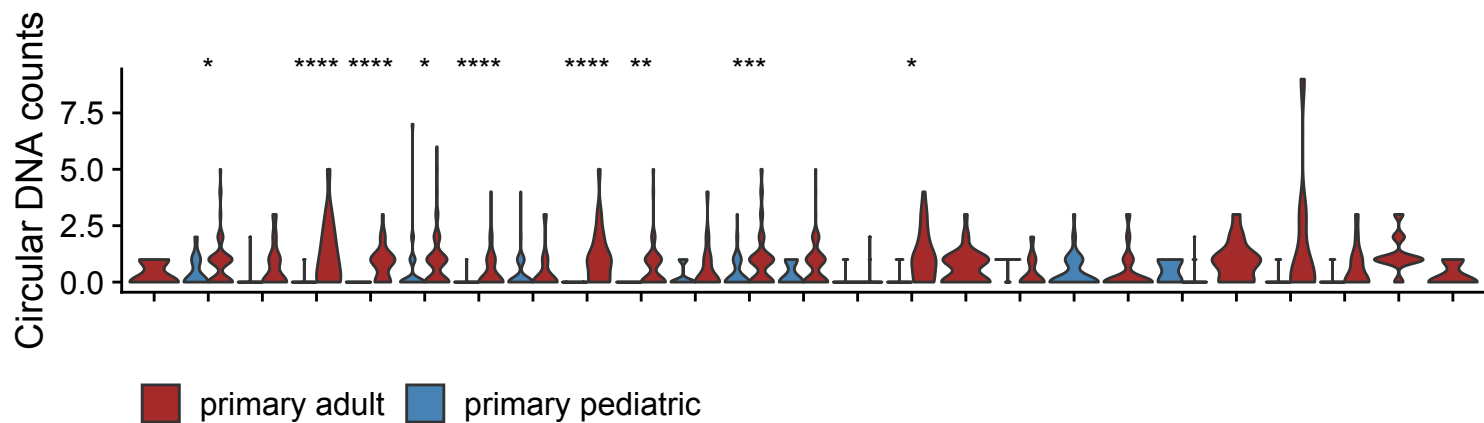

C

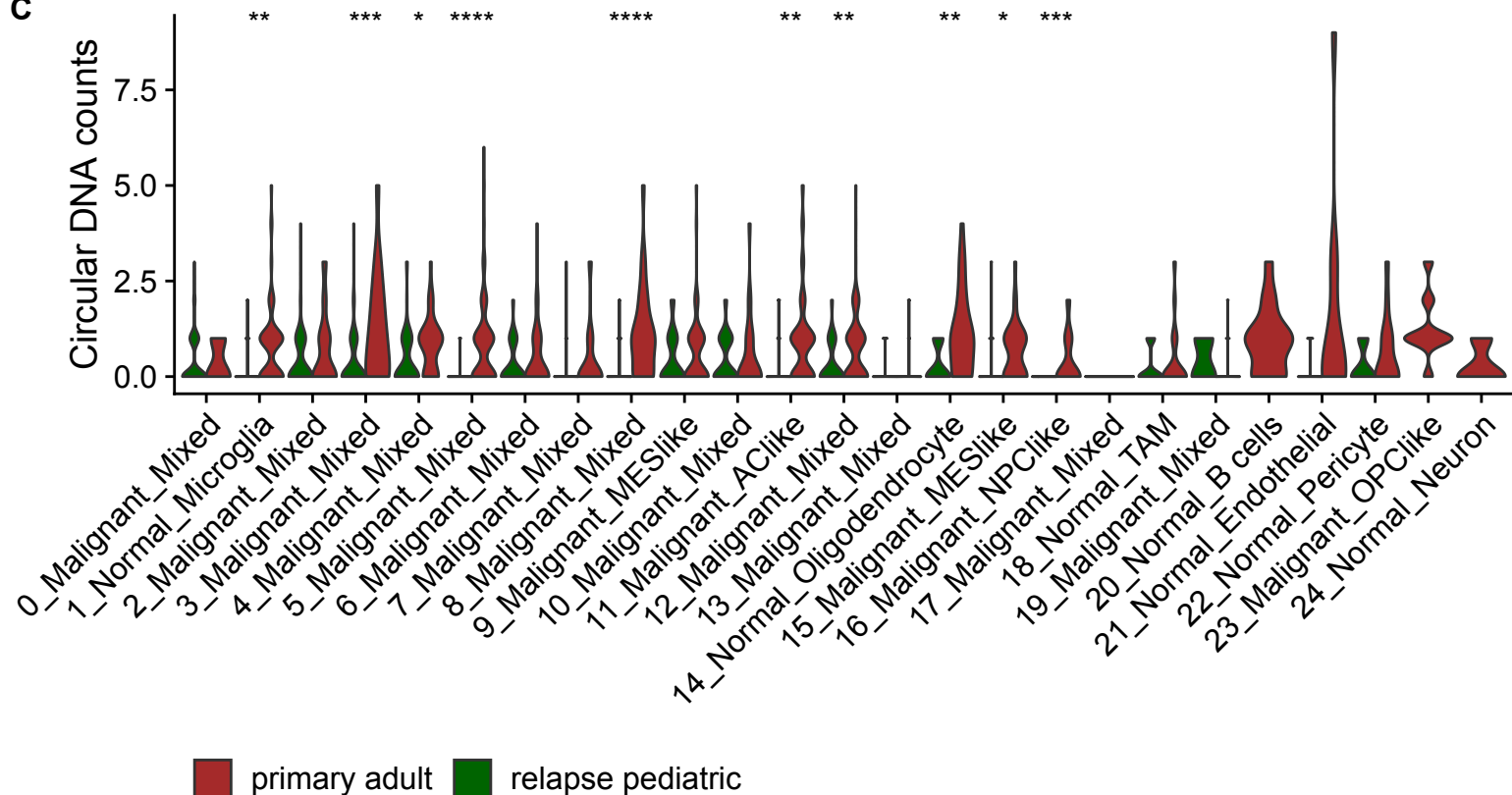

Supplement: Supplementary file 3 — Supplementary material [file mmc3.pdf]
