# Supplementary material for: Investigating cellular heterogeneity at the single-cell level by the flexible and mobile extrachromosomal circular DNA
Source: Comput Struct Biotechnol J. 2023 Jan 24;21:1115–21. doi: 10.1016/j.csbj.2023.01.025 (PMC9900259; doi:10.1016/j.csbj.2023.01.025)
Supplement: Supplementary file 4 — Supplementary material [file mmc4.pdf]

ST 1 Sample information

GEO accession ID	Sample ID	Condition	Sex	Age (years)	Histology	Primary or Recurrent	Genetic alterations
GSE139136	GSM4131776	Adult	Male	64	Glioblastoma	Primary	Wild type for IDH1 and IDH2
	GSM4131777	Adult	Male	73	Glioblastoma	Primary	Wild type for IDH1 and IDH2
	GSM4131778	Adult	Female	52	Glioblastoma	Primary	Wild type for IDH1 and IDH2
	GSM4131779	Adult	Male	62	Glioblastoma	Primary	Wild type for IDH1 and IDH2
GSE163655	GSM4983564	Pediatric	n/a	n/a	Glioblastoma	Primary	n/a
	GSM4983566	Pediatric	n/a	n/a	Glioblastoma	Primary	n/a
GSE163656	GSM4983567	Pediatric recurrent	n/a	n/a	Glioblastoma	Recurrent	n/a
	GSM4983568	Pediatric recurrent	n/a	n/a	Glioblastoma	Recurrent	n/a
	GSM4983569	Pediatric recurrent	n/a	n/a	Glioblastoma	Recurrent	n/a

n/a: not available.
