# Supplementary material for: Investigating cellular heterogeneity at the single-cell level by the flexible and mobile extrachromosomal circular DNA
Source: Comput Struct Biotechnol J. 2023 Jan 24;21:1115–21. doi: 10.1016/j.csbj.2023.01.025 (PMC9900259; doi:10.1016/j.csbj.2023.01.025)
Supplement: Supplementary file 5 — Supplementary material [file mmc5.pdf]

Supplementary Information

Investigating cellular heterogeneity at the single-cell level by the flexible and mobile extrachromosomal circular DNA

Table of contents

1. ST 1. Sample information
2. Supplementary Figures 1-3

ST 1. Sample information

ST 1 Sample information

GEO accession ID	Sample ID	Condition	Sex	Age (years)	Histology	Primary or Recurrent	Genetic alterations
GSE139136	GSM4131776	Adult	Male	64	Glioblastoma	Primary	Wild type for IDH1 and IDH2
	GSM4131777	Adult	Male	73	Glioblastoma	Primary	Wild type for IDH1 and IDH2
	GSM4131778	Adult	Female	52	Glioblastoma	Primary	Wild type for IDH1 and IDH2
	GSM4131779	Adult	Male	62	Glioblastoma	Primary	Wild type for IDH1 and IDH2
GSE163655	GSM4983564	Pediatric	n/a	n/a	Glioblastoma	Primary	n/a
	GSM4983566	Pediatric	n/a	n/a	Glioblastoma	Primary	n/a
GSE163656	GSM4983567	Pediatric recurrent	n/a	n/a	Glioblastoma	Recurrent	n/a
	GSM4983568	Pediatric recurrent	n/a	n/a	Glioblastoma	Recurrent	n/a
	GSM4983569	Pediatric recurrent	n/a	n/a	Glioblastoma	Recurrent	n/a

n/a: not available.

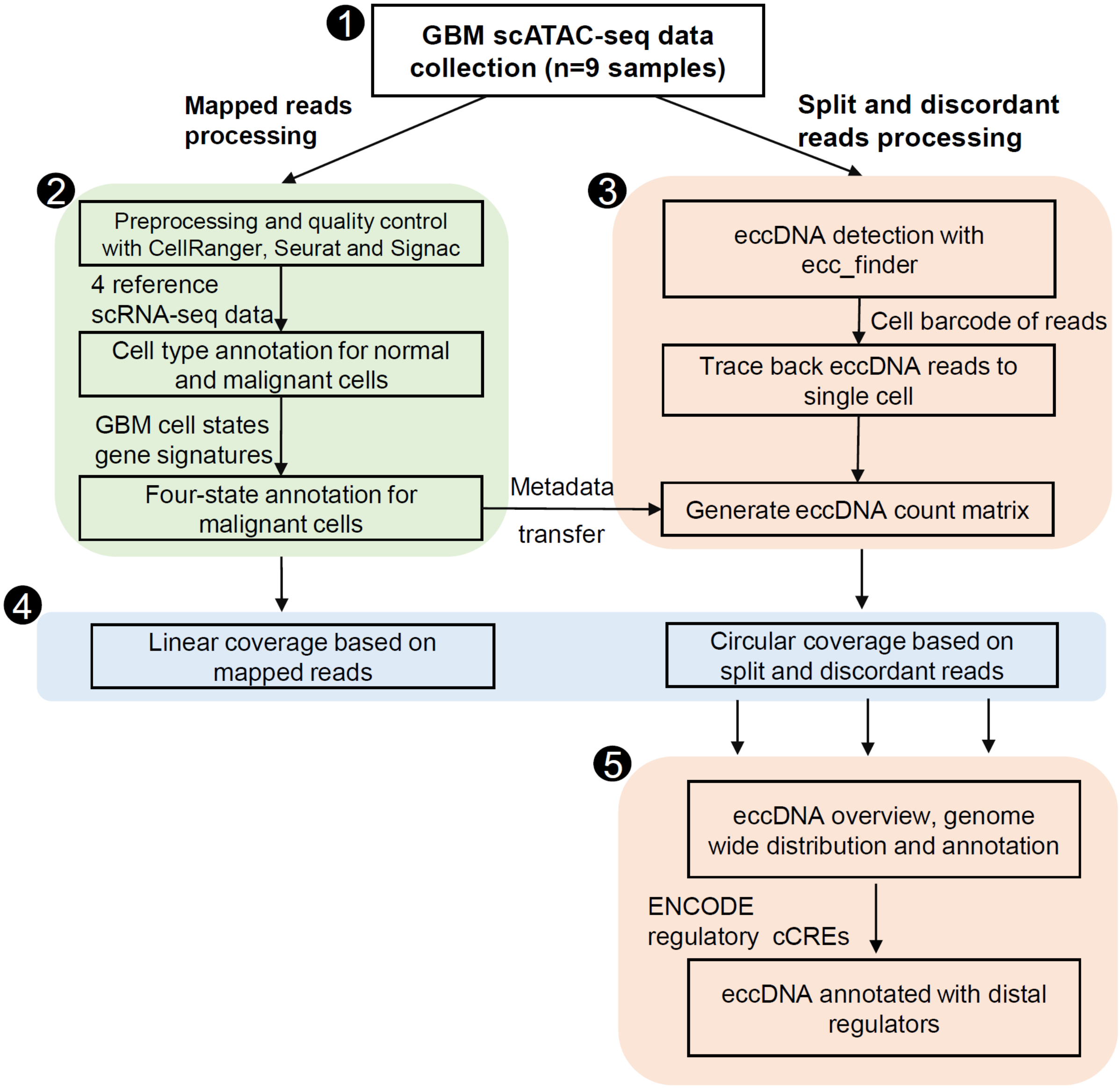

SF 1. The methodological framework of eccDNA identification. Step 1: scATAC-seq data collection. The data from a total of 9 GBM samples were collected. Step 2: Process and annotate the GBM scATAC-seq data based on mapped reads. Step 3: eccDNA detection and their single-cell origin traceback using ecc_finder based on split and discordant reads. Step 4: Generate circular and linear coverage of common eccDNAs based on eccDNA count matrix and annotated scATAC-seq data. Step 5: Provide the genome distribution overview and annotation of eccDNA mapped regions.

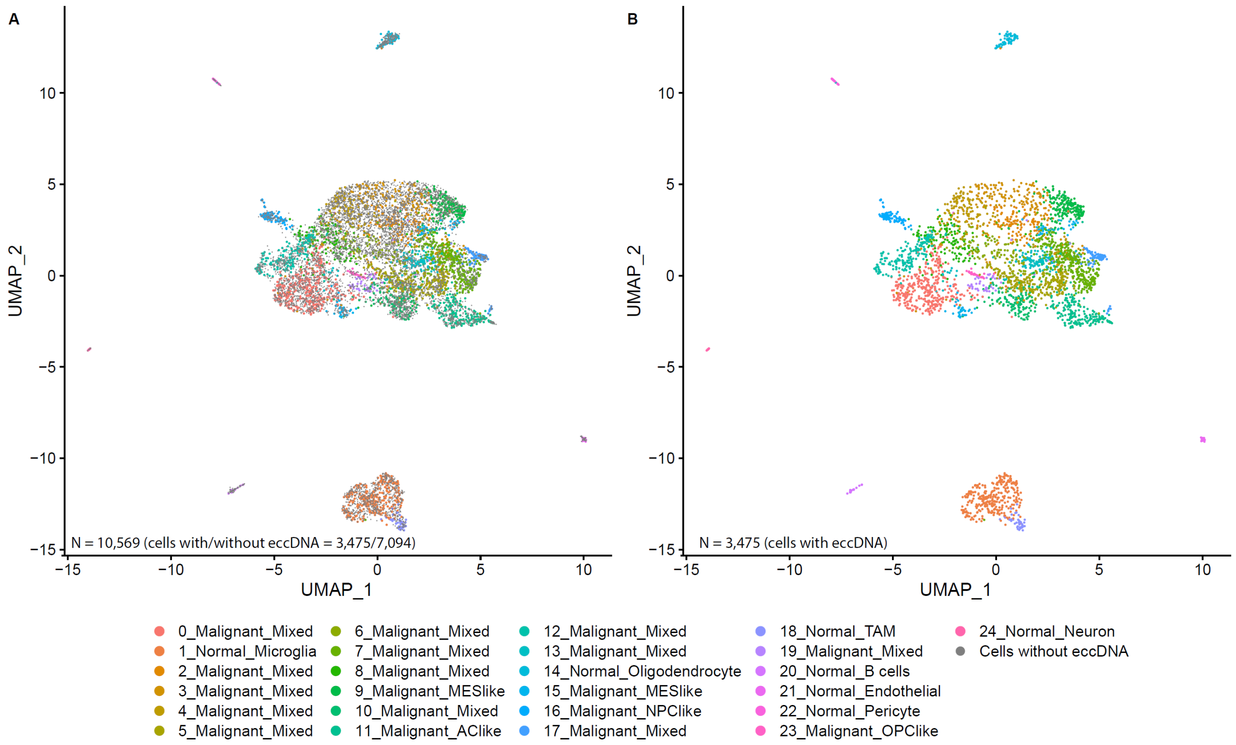

SF 2. Overview of cell clusters in the integrated scATAC-seq GBM data. A. UMAP of all cells passing quality controls. Cells harboring eccDNAs are colored by annotated cell types and cell states. Cells without eccDNAs are colored in grey. **B.** UMAP of cells with eccDNA colored by annotated cell types and cell states.

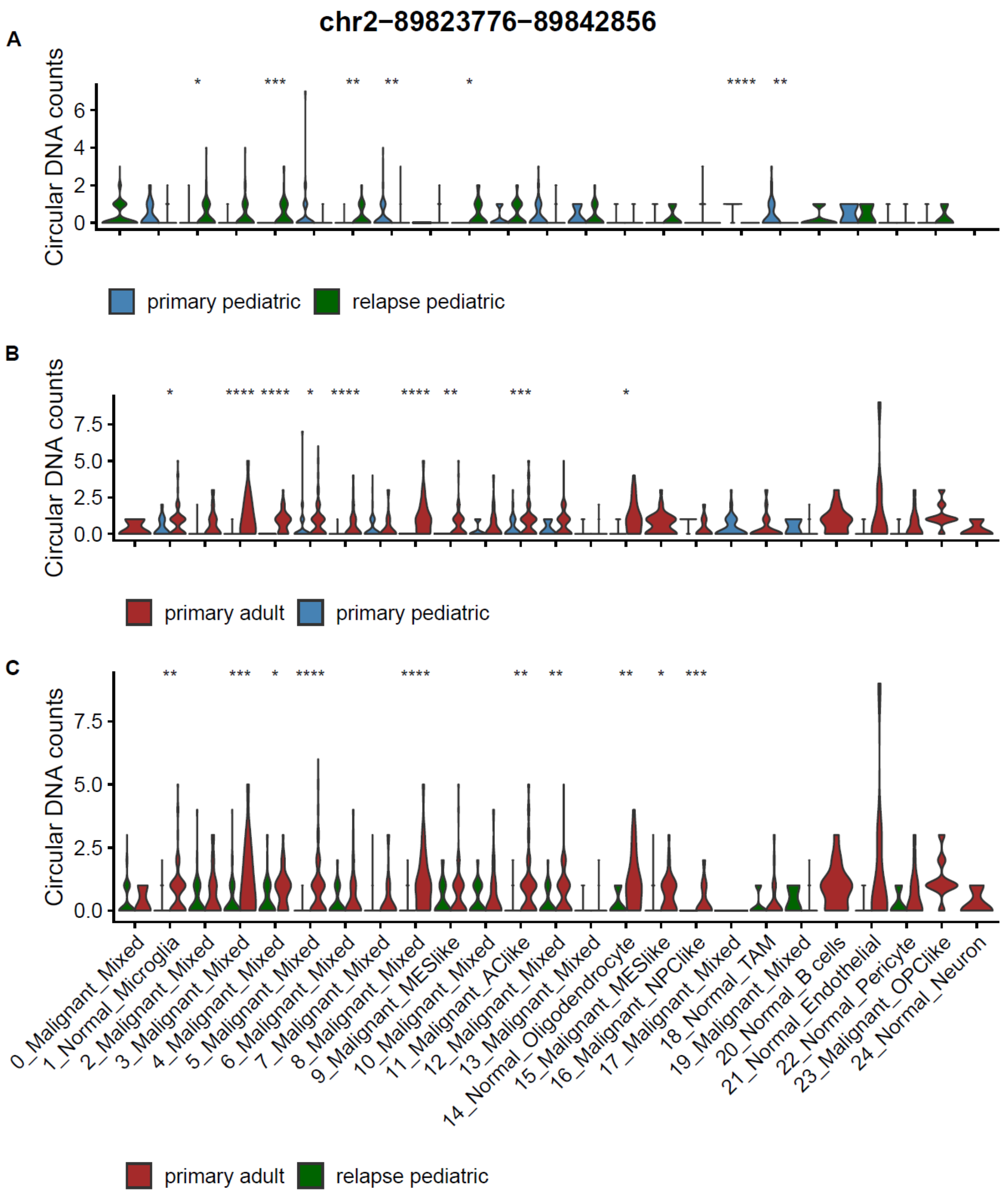

SF 3. Condition-wise comparison of circular coverage of eccDNA at the locus chr2:89823776−89842856. **A.** Comparison between primary pediatric and relapse pediatric GBMs. **B.** Comparison between primary adult and primary pediatric GBMs. **C.** Comparison between primary adult and relapse pediatric GBMs. Cell cluster names plotted on the x-axis are organized in the format of [Cluster ID]_[Tumor or normal cell]_[cell type or cell states]. Mixed: the

malignant cluster is a mixture of at least two cell states out of MESlike, OPClike, NPClike and AClike. Abbreviations: TAM: tumor-associated macrophage; NPClike, neural-progenitor-like; OPClike, oligodendrocyte-progenitor-like; AClike, astrocyte-like; MESlike, mesenchymal-like. Wilcox test was applied to compare the eccDNA distribution in each cell type between two conditions in each pair. ****: $p < 0.0001$. ***: $p < 0.001$. **: $p < 0.01$. *: $p < 0.05$.
